# Automated 3D segmentation of the aorta and pulmonary artery for predicting outcomes after thoracoscopic lobectomy in lung cancer patients

**DOI:** 10.3389/fonc.2022.1027036

**Published:** 2022-10-28

**Authors:** Hsin-Ying Lee, Yu-Jung Chung, Hao-Jen Wang, Xu-Heng Chiang, Li-Wei Chen, Yan-Ting Lin, Yi-Chieh Lee, Hsao-Hsun Hsu, Yeun-Chung Chang, Chung-Ming Chen, Mong-Wei Lin, Jin-Shing Chen

**Affiliations:** ^1^ Department of Medicine, College of Medicine, National Taiwan University, Taipei, Taiwan; ^2^ Institute of Biomedical Engineering, College of Medicine and College of Engineering, National Taiwan University, Taipei, Taiwan; ^3^ Institute of Epidemiology and Preventive Medicine, College of Public Health, National Taiwan University, Taipei, Taiwan; ^4^ Department of Surgery, National Taiwan University Hospital, Taipei, Taiwan; ^5^ Department of Medical Imaging , National Taiwan University Hospital and National Taiwan University College of Medicine, Taipei, Taiwan; ^6^ Department of Surgical Oncology, National Taiwan University Cancer Center, Taipei, Taiwan

**Keywords:** aorta, computed tomography, lobectomy, lung cancer, pulmonary artery, pulmonary hypertension, segmentation

## Abstract

**Background:**

Preoperative two-dimensional manual measurement of pulmonary artery diameter in a single-cut axial view computed tomography (CT) image is a commonly used non-invasive prediction method for pulmonary hypertension. However, the accuracy may be unreliable. Thus, this study aimed to evaluate the correlation of short-term surgical outcomes and pulmonary artery/aorta (PA/Ao) diameter ratio measured by automated three-dimensional (3D) segmentation in lung cancer patients who underwent thoracoscopic lobectomy.

**Materials and methods:**

We included 383 consecutive lung cancer patients with thin-slice CT images who underwent lobectomy at a single institute between January 1, 2011 and December 31, 2019. Automated 3D segmentation models were used for 3D vascular reconstruction and measurement of the average diameters of Ao and PA. Propensity-score matching incorporating age, Charlson comorbidity index, and lobectomy performed by uniportal VATS was used to compare clinical outcomes in patients with PA/Ao ratio ≥1 and those <1.

**Results:**

Our segmentation method measured 29 (7.57%) patients with a PA/Ao ratio ≥1. After propensity-score matching, a higher overall postoperative complication classified by the Clavien–Dindo classification (p = 0.016) were noted in patients with 3D PA/Ao diameter ratio ≥1 than those of <1. By multivariate logistic regression, patients with a 3D PA/Ao ratio ≥ 1 (p = 0.013) and tumor diameter > 3 cm (p = 0.002) both significantly predict the incidence of postoperative complications.

**Conclusions:**

Pulmonary artery/aorta diameter ratio ≥ 1 measured by automated 3D segmentation may predict postoperative complications in lung cancer patients who underwent lobectomy.

## Introduction

Pulmonary lobectomy remains the standard treatment for early lung cancer (1). With rapid advancements in operation techniques, video-assisted thoracoscopic surgery (VATS) has been shown to cause fewer postoperative complications and a shorter hospital length of stay than conventional thoracotomy (2–5). However, despite an adequate preoperative assessment, complications may still appear after VATS lobectomy.

The presence of pulmonary hypertension (PH) has been reported to be an indicator of postoperative complications (6, 7). Nevertheless, confirmation of PH by catheterization or echocardiography is labor-intensive, time-consuming, and subject to inter-rater variability (6, 8). Consequently, considering the relationship between the elevated pulmonary artery (PA) pressure and size, recent studies have linked an enlarged PA with postoperative complications (9–11). However, previous studies used two-dimensional (2D) measurements on single-cut axial view contrast-enhanced computed tomography (CT) images. Thus, automatic three-dimensional (3D) segmentation methods to accurately calculate the mean 3D diameters of both the aorta (Ao) and PA on CT images have not been reported before.

Furthermore, with the emphasis on screening programs, lung cancer diagnosis on non-contrast CT has increased significantly (12). While this approach involves minimal radiocontrast exposure, it poses challenges in differentiating blood pool regions and surrounding tissues. Prior studies developed an automatic segmentation method to overcome the difficulty in identifying Ao; however, similar analyses have not been conducted on PA (13, 14). Our team previously developed a novel automated 3D segmentation method for both Ao and PA on non-contrast CT images (15). The proposed model may achieve a high level of segmentation performance, including 0.97 ± 0.007 and 0.93 ± 0.002 Dice similarity coefficient values for Ao and PA segmentation, respectively.

The present study aimed to evaluate the relationship between the PA/Ao diameter ratio measured by automated 3D segmentation and short-term surgical outcomes after thoracoscopic lobectomy in lung cancer patients.

## Materials and methods

### Study population

**Figure 1:**
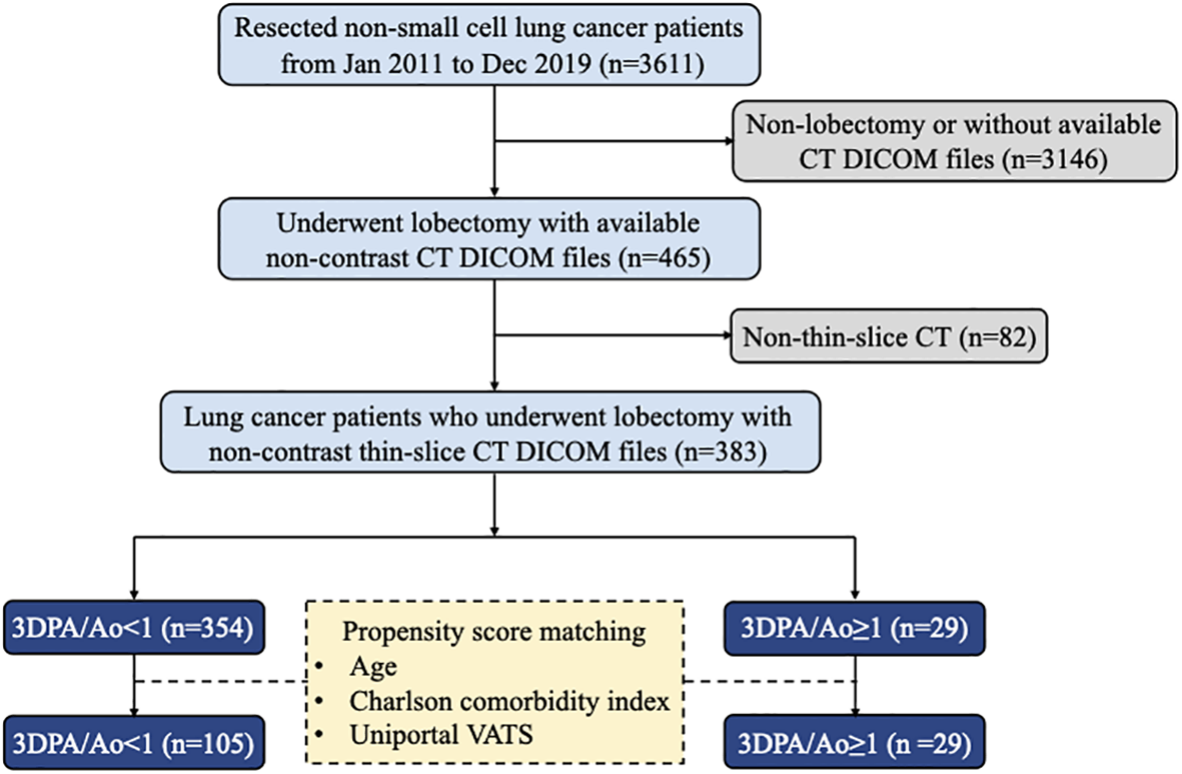
Flow diagram of patient selection.

In this retrospective study, we collected clinical data and CT scans from 465 consecutive primary lung cancer patients who underwent thoracoscopic lobectomy performed by a single surgical team using the same clinical protocols, care patterns, and perioperative orders at the National Taiwan University Hospital between January 2011 and December 2019. Cases with non-thin-slice CT scans were excluded from our study. The final cohort consisted of 383 patients grouped according to the ratio of the PA and Ao diameters. The patient inclusion and exclusion flow diagram is demonstrated in **Figure 1**. The Research Ethics Committee of the National Taiwan University Hospital approved this study (project approval number 201712087RIND) and waived the need for obtaining informed consent because of the retrospective study design.

### Clinicopathological features

Clinical data, including patient demographics, Eastern Cooperative Oncology Group performance status, family history of lung cancer, preoperative carcinoembryonic antigen level, preoperative pulmonary function test results, operation time, operative bleeding, postoperative chest tube placement duration, and hospital length of stay, were retrieved from our electronic medical records. Preoperative comorbidities were evaluated according to the Charlson comorbidity index (16). Postoperative complications were graded by the Clavien–Dindo classification (17). For patients presenting with multiple complications, the complication with the highest grade was adopted.

Information on tumor size, predominant histological type, degree of differentiation, visceral pleural invasion, lymphovascular invasion, pathological T and N stages, number of dissected lymph nodes, number of dissected lymph node stations, and resection margins were collected from pathological reports of operation specimens. Histopathological patterns were classified according to the 2015 World Health Organization criteria (18). Lung cancer staging was determined based on the eighth edition of the American Joint Committee on Cancer Tumor-Node-Metastasis staging system (19).

### CT acquisition

Pulmonary CT images were obtained using scanners from GE (LightSpeed VCT, LightSpeed 16, and HiSpeed CT/i), Siemens (Definition AS+, Emotion 16, and Sensation 64), and Philips (iCT 256 and Ingenuity CT) Healthcare systems. The CT image parameters were as follows: 100–130 kVp; 47–351 mA; slice thickness, 0.6–1.25 mm; pixel spacing, 0.38–0.89 mm; reconstruction interval, 0.39–6 mm; and matrix, 512 mm × 512 mm.

### Manual 2D PA/Ao diameter ratio measurement

**Figure 2:**
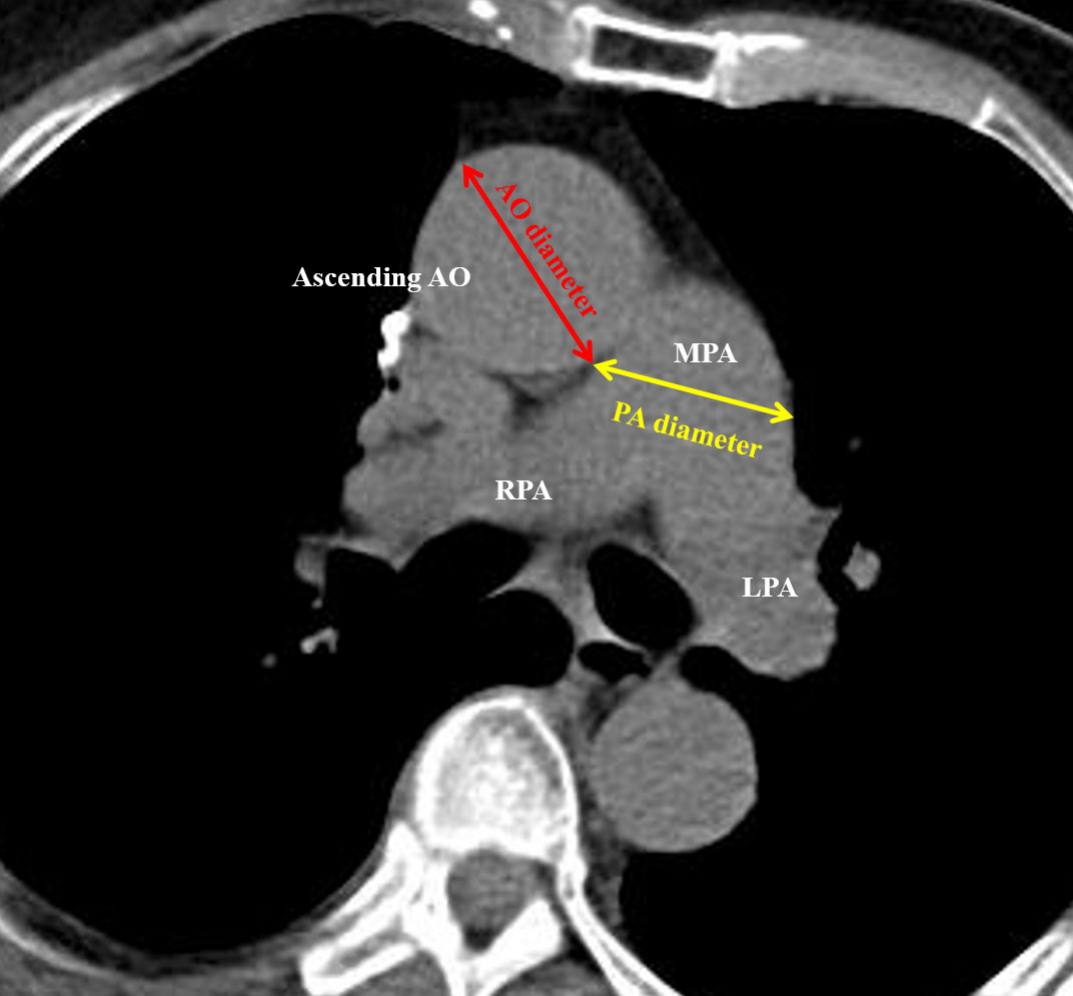
Illustration of 2D manual measurement of the pulmonary artery (PA) and aorta (Ao) diameters.

A total of 383 CT scans were annotated by two of four physicians (HY Lee, XH Chiang, YC Lee, and MW Lin) blinded the patient’s postoperative course, and recorded after a consensus was reached. The PA and Ao diameters were determined on non-contrast CT. As previously reported, measurements were conducted in the axial view at the level of the main PA bifurcation, which generally represents the widest point of Ao. We then assessed the diameters of PA and Ao on the same image and calculated the PA/Ao diameter ratio as an indicator of PA enlargement. An example of manual 2D annotation is illustrated in **Figure 2**.

### Automated 3D segmentation and PA/Ao diameter ratio measurement

**Figure 3:**
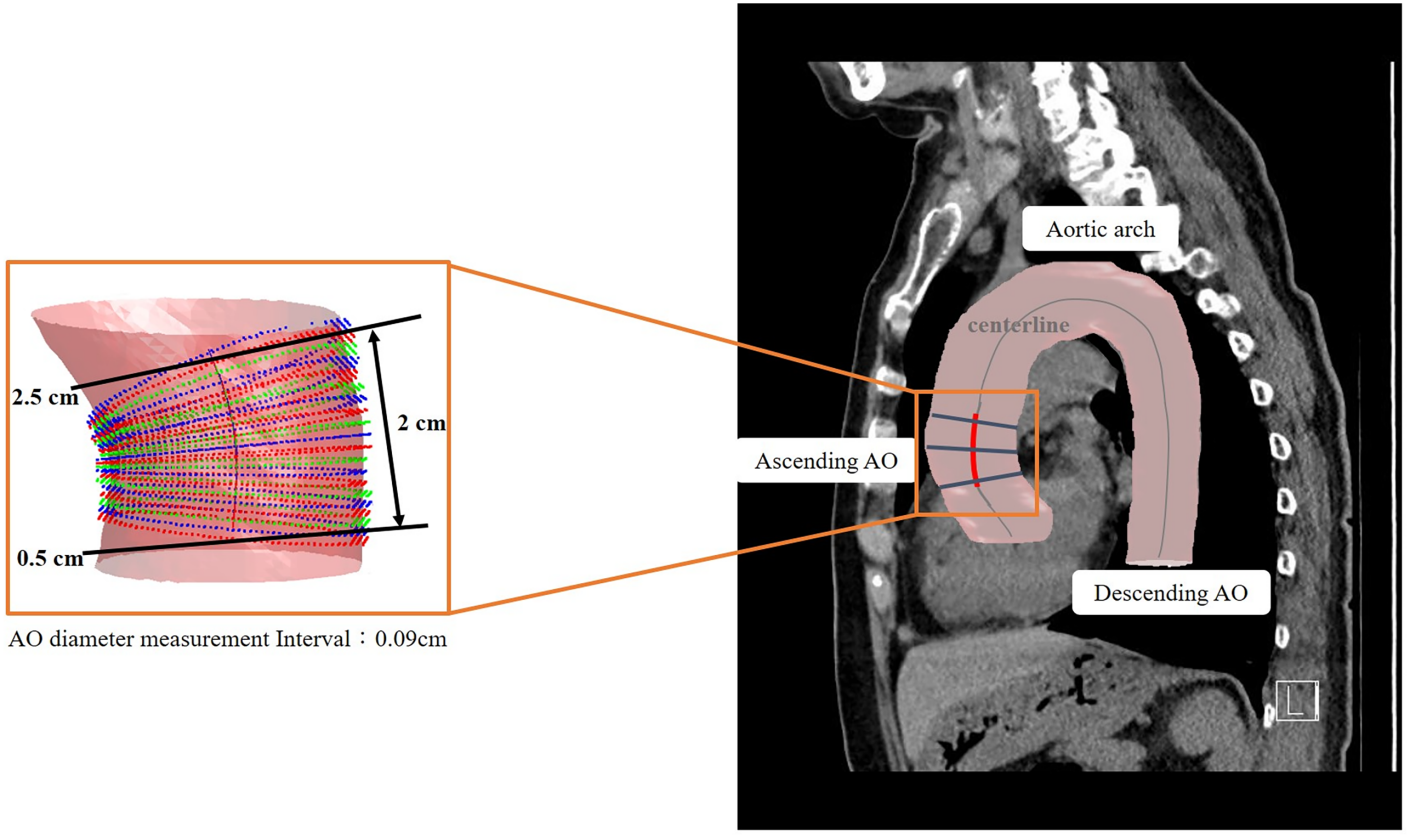
Illustration of 3D automated measurement of aorta (Ao) diameter. The centerline was marked on the 3D segmented image. The Ao diameter was measured from 0.5 cm after exiting the heart to a position of 2.5 cm. The Ao diameter was defined as the mean value of calculated diameters at every 0.04-cm interval.

**Figure 4:**
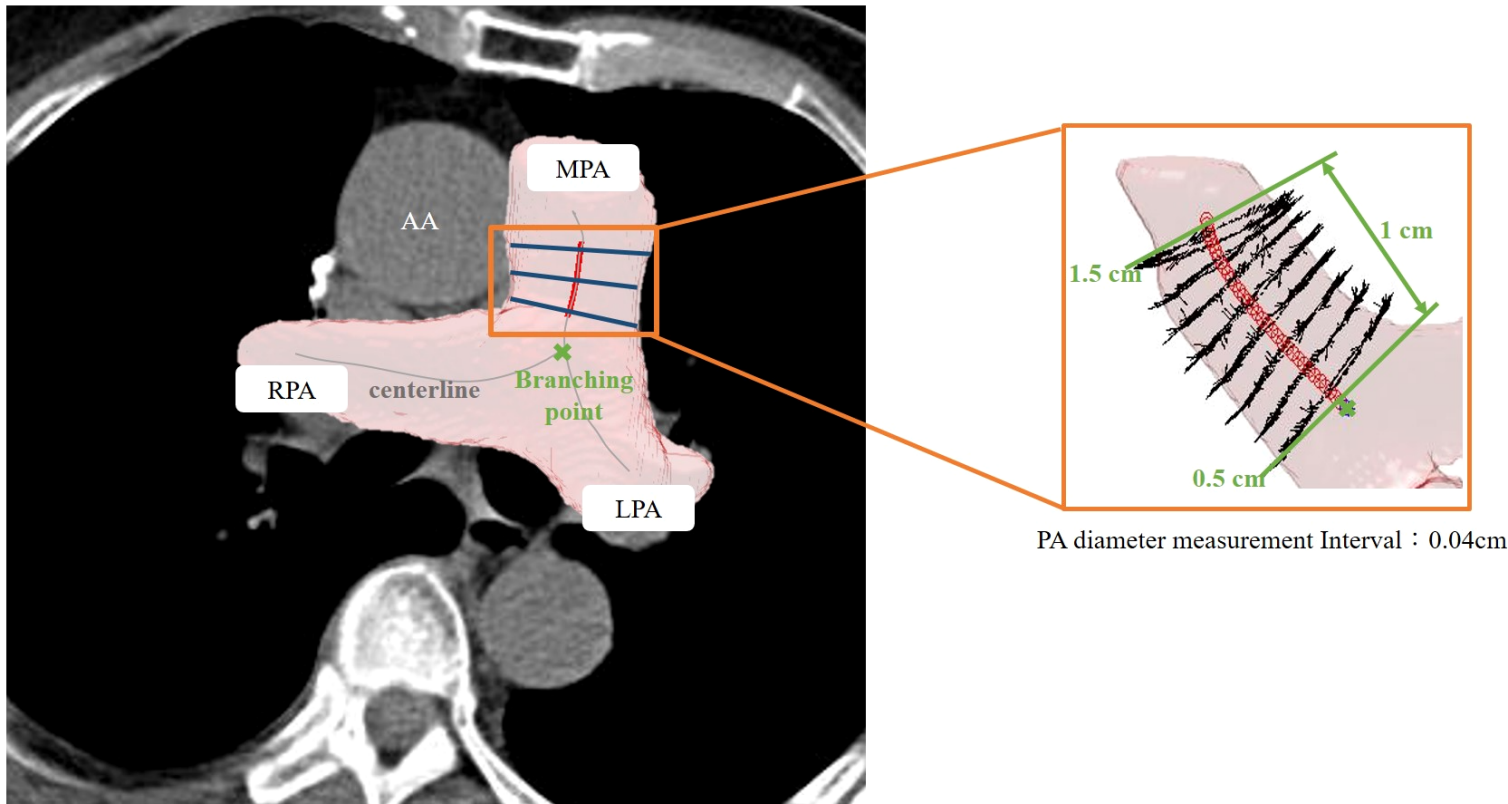
Illustration of 3D automated measurement of pulmonary artery (PA) diameter. The centerline was marked on the 3D segmented image. The PA diameter was measured on the main PA between 0.5 to 1.5 cm from the branching point. The PA diameter was defined as the mean value of calculated diameters at every 0.09-cm interval.

The details of the two-stage automated 3D PA and Ao segmentation model have been fully described in a previous study (15). In summary, both models applied the transfer learning from the enhancement model. Single non-contrast CT was used as the input for Ao segmentation, whereas an additional two-channel input was employed for PA. For Ao, the diameter was calculated from 0.5 cm after exiting the heart to the position of 2.5 cm, with the diameter estimated at 0.09 cm intervals. For PA, the diameter was measured on the main PA, between 0.5 to 1.5 cm, from the branching point. The PA diameter was calculated at 0.04 cm intervals. Examples of 3D annotation are illustrated in **Figures 3** and **4**.

### Statistical analysis

Categorical variables were presented as numbers (with percentages), while continuous variables were presented as means (with standard deviation). Chi-square test or Fisher’s exact test was performed for categorical variables. Student’s t-test or Mann–Whitney U test was utilized for continuous variables according to the normality. Spearman correlation was carried out to account for the relationship between 3D automated and 2D manual measurements of PA and Ao. To adjust the significant confounding factors in the univariate model, a multivariable analysis was performed by logistic regression. Propensity-score matching (PSM) was employed to minimize selection bias. The propensity score was calculated using a logistic regression model based on the following covariates: age, Charlson comorbidity score, and lobectomy performed by uniportal VATS. Patients in the PA enlarged group (PA/Ao ≥ 1) were matched with those in the PA non-enlarged group (PA/Ao < 1) in a 1:4 ratio and a standard deviation of <0.1 of the logit propensity score. All p values were two-sided, and p < 0.05 was considered statistically significant. The statistical software used for all analyses was SPSS (add-on for SPSS, version 3.04; IBM Corp., Armonk, NY) with the underlying R package (version 3.3.0; R Foundation, Vienna, Austria) and Statistical Analysis System version 9.4 software (SAS Institute Inc., Cary, NC, USA).

## Results

### Patient demographics and clinicopathological features

This study cohort contains 383 patients who were diagnosed with lung cancer and underwent lobectomy between 2011 and 2019. Of these, 29 (7.57%) patients presented with a PA/Ao diameter ≥ 1 on non-contrast CT by 3D automatic measurement. Only age and the Charlson comorbidity index were significantly different between patients with 3D-measured PA/Ao ≥ 1 and PA/Ao < 1. Patients with 3D-measured PA/Ao ≥ 1 were younger (54.4 ± 9.3 *vs*. 61.0 ± 9.7 years; p = 0.001) and showed a lower Charlson comorbidity index (1.7 ± 1.5 *vs*. 2.3 ± 1.7; p = 0.029). Patient demographics and clinical characteristics are presented in **Table 1**.

**Table 1:** Demographic and clinical features of lung cancer patients undergoing lobectomy.

	Before matching				After matching			
	All (n = 383)	3DPA/Ao<1 (n = 354)	3DPA/Ao≥1 (n = 29)	p value	All (n = 134)	PA/Ao<1 (n = 105)	PA/Ao≥1 (n = 29)	p value
Age, years	60.5 ± 9.8	61.0 ± 9.7	54.4 ± 9.3	0.001	55.5 ± 9.6	55.8 ± 9.7	54.4 ± 9.3	0.504
Female	242 (63.2%)	226 (63.8%)	16 (55.2%)	0.352	81 (60.4%)	65 (61.9%)	16 (55.2%)	0.512
Smoking	76 (19.8%)	72 (20.3%)	4 (13.8%)	0.395	22 (16.4%)	18 (17.1%)	4 (13.8%)	0.783
ECOG				0.391				0.659
0	292 (76.2%)	268 (75.7%)	24 (82.8%)		107 (79.9%)	83 (79.0%)	24 (82.8%)	
≥1	91 (23.8%)	86 (24.3%)	5 (17.2%)		27 (20.1%)	22 (21.0%)	5 (17.2%)	
PFT								
FVC, %^a^	108.7 ± 14.7	108.8 ± 14.8	107.6 ± 13.3	0.778	107.6 ± 13.3	107.5 ± 13.4	107.6 ± 13.3	0.974
FEV1, %^a^	108.5 ± 17.6	108.4 ± 17.9	109.9 ± 14.0	0.542	106.0 ± 16.1	105.0 ± 16.5	109.9 ± 14.0	0.156
Lung cancer family history	71 (18.5%)	66 (18.6%)	5 (17.2%)	0.852	22 (16.4%)	17 (16.2%)	5 (17.2%)	>0.999
Comorbidity index (CCI)	2.3 ± 1.7	2.3 ± 1.7	1.7 ± 1.5	0.029	1.9 ± 1.7	1.9 ± 1.7	1.7 ± 1.5	0.484
CEA level^a^				>0.999				>0.999
Normal	308 (86.8%)	284 (86.6%)	24 (88.9%)		110 (86.6%)	86 (86.0%)	24 (88.9%)	
Abnormal^b^	47 (13.2%)	44 (13.4%)	3 (11.1%)		17 (13.4%)	14 (14.0%)	3 (11.1%)	

Data are presented as mean ± standard deviation or number (%).

3DPA/Ao, Pulmonary artery/aorta diameter ratio measured on 3D images; CCI, Charlson comorbidity index; CEA, carcinoembryonic antigen; ECOG, Eastern Cooperative Oncology Group performance status; FEV1, forced expiratory volume in 1 second; FVC, forced vital capacity; PA/Ao, ratio of the diameters of the pulmonary artery and aorta; PFT, pulmonary function test.

a Missing value for FVC/FEV1 (n = 10) and CEA level (n = 28).

b An abnormal CEA level was defined as ≥ 5 ng/mL.

Patients with 3D-measured PA/Ao ≥ 1 and <1 showed no significant differences in pathological features. The most common histological type in both groups was adenocarcinoma (3D PA/Ao ≥ 1: 96.6%; 3D PA/Ao < 1: 95.8%). Among patients with 3D measured PA/Ao ≥ 1, 86.2% had a lung tumor of ≤3 cm, and 58.6% belonged to stage I. Detailed information on the pathological features is listed in **Table 2**.

**Table 2:** Pathological features of the excised tumors of lung cancer patients undergoing lobectomy.

	Before matching				After matching			
	All (n = 383)	3DPA/Ao<1 (n = 354)	3DPA/Ao≥1 (n = 29)	p value	All (n = 134)	PA/Ao<1 (n = 105)	PA/Ao≥1 (n = 29)	p value
Histology				>0.999				>0.999
Non-adenocarcinoma^a^	16 (4.2%)	15 (4.2%)	1 (3.4%)		7 (5.2%)	6 (5.7%)	1 (3.4%)	
Adenocarcinoma	367 (95.8%)	339 (95.8%)	28 (96.6%)		127 (94.8%)	99 (94.3%)	28 (96.6%)	
Differentiation				0.273				0.919
Poor^b^	53 (14.1%)	47 (13.5%)	6 (20.7%)		28 (21.4%)	22 (21.6%)	6 (20.7%)	
Well and moderate	323 (85.9%)	300 (86.5%)	23 (79.3%)		103 (78.6%)	80 (78.4%)	23 (79.3%)	
VPI-positive	78 (20.4%)	73 (20.6%)	5 (17.2%)	0.664	24 (17.9%)	19 (18.1%)	5 (17.2%)	0.915
LVI-positive	73 (19.1%)	69 (19.5%)	4 (13.8%)	0.453	29 (21.6%)	25 (23.8%)	4 (13.8%)	0.246
Pathological tumor size, cm				0.879				0.658
≤3	328 (85.6%)	303 (85.6%)	25 (86.2%)		117 (87.3%)	92 (87.6%)	25 (86.2%)	
3-5	47 (12.3%)	43 (12.1%)	4 (13.8%)		14 (10.4%)	10 (9.5%)	4 (13.8%)	
5-7	8 (2.1%)	8 (2.3%)	0 (0.0%)		3 (2.2%)	3 (2.9%)	0 (0%)	
>7	0 (0%)	0 (0%)	0 (0.0%)		0 (0%)	0 (0%)	0 (0.0%)	
N stage				0.356				0.732
N0	330 (86.2%)	307 (86.7%)	23 (79.3%)		108 (80.6%)	85 (81.0%)	23 (79.3%)	
N1	22 (5.7%)	20 (5.6%)	2 (6.9%)		12 (9.0%)	10 (9.5%)	2 (6.9%)	
N2	31 (8.1%)	27 (7.6%)	4 (13.8%)		14 (10.4%)	10 (9.5%)	4 (13.8%)	
Pathological stage				0.361				0.724
IA	243 (63.4%)	226 (63.8%)	17 (58.6%)		85 (63.4%)	68 (64.8%)	17 (58.6%)	
IB	22 (5.7%)	22 (6.2%)	0 (0.0%)		3 (2.2%)	3 (2.9%)	0 (0.0%)	
II	84 (21.9%)	76 (21.5%)	8 (27.6%)		32 (23.9%)	24 (22.9%)	8 (27.6%)	
III	34 (8.9%)	30 (8.5%)	4 (13.8%)		14 (10.4)	10 (9.5%)	4 (13.8%)	
IV	0 (0.0%)	0 (0.0%)	0 (0.0%)		0 (0.0%)	0 (0.0%)	0 (0.0%)	
Resection margin				0.678				0.610
Negative	361 (94.3%)	334 (94.4%)	27 (93.1%)		128 (95.5%)	101 (96.2%)	27 (93.1%)	
Positive	22 (5.7%)	20 (5.6%)	2 (6.9%)		6 (4.5%)	4 (3.8%)	2 (6.9%)	

Data are presented as mean ± standard deviation or number (%).

3DPA/Ao, Pulmonary artery/aorta diameter ratio measured on 3D images; LVI, lymphovascular invasion; PA/Ao, ratio of the diameters of the pulmonary artery and aorta; VPI, visceral pleura invasion.

a Non-adenocarcinoma includes squamous cell carcinoma (n = 6), adenosquamous carcinoma (n = 3), small and squamous carcinoma (n = 1), carcinoid tumor (n = 2), lymphoepithelial-like carcinoma (n = 1), pleomorphic carcinoma (n = 1), sarcomatoid carcinoma (n = 1), and unspecified (n = 1).

b Missing value for differentiation (n = 7).

### Perioperative outcomes before matching

The 30-day mortality in the study cohort was zero, and only two patients in the 3D-measured PA/Ao < 1 group converted from VATS to thoracotomy. Patients with 3D-measured PA/Ao ≥ 1 required a longer intensive care unit (1.4 ± 5.3 *vs*. 0.3 ± 0.8 days; p = 0.805) and postoperative hospital stays (6.9 ± 9.0 *vs*. 5.7 ± 3.6 days; p = 0.403), although the differences were not significant, while operative bleeding (34.3 ± 58.8 *vs*. 42.5 ± 106.7 mL) and chest tube placement duration (2.6 ± 1.9 *vs*. 3.2 ± 3.2 days) were non-significantly lesser and shorter in patients with 3D-measured PA/Ao ≥ 1. Other perioperative outcomes are presented in **Table 3**.

**Table 3:** Perioperative outcomes of lung cancer patients undergoing lobectomy.

	Before matching				After matching			
	All (n = 383)	3DPA/Ao<1 (n = 354)	3DPA/Ao≥1 (n = 29)	p value	All (n = 134)	PA/Ao<1 (n = 105)	PA/Ao≥1 (n = 29)	p value
Approach method				>0.999				>0.999
Thoracotomy	0 (0.0%)	0 (0.0%)	0 (0.0%)		0 (0.0%)	0 (0.0%)	0 (0.0%)	
VATS	383 (100%)	354 (100%)	29 (100%)		134 (100%)	105 (100%)	29 (100%)	
Conversion to thoracotomy	2 (0.5%)	2 (0.6%)	0 (0.0%)	>0.999	0 (0.0%)	0 (0.0%)	0 (0.0%)	>0.999
Dissected LNs								
Total number	13.8 ± 7.5	13.8 ± 7.6	13.7 ± 6.3	0.852	14.0 ± 7.3	14.2 ± 7.5	13.7 ± 6.3	0.758
Total station	4.6 ± 1.3	4.6 ± 1.3	4.7 ± 1.3	0.781	4.6 ± 1.3	4.6 ± 1.3	4.7 ± 1.3	0.724
Non-intubated anesthesia	103 (26.9%)	95 (26.8%)	8 (27.6%)	0.930	40 (29.9%)	32 (30.5%)	8 (27.6%)	0.763
Uniportal VATS	112 (29.2%)	99 (28.0%)	13 (44.8%)	0.055	55 (41.0%)	42 (40.0%)	13 (44.8%)	0.640
Operative bleeding, mL	41.9 ± 103.8	42.5 ± 106.7	34.3 ± 58.8	0.542	31.0 ± 79.6	30.1 ± 84.7	34.3 ± 58.8	0.806
Post-op ICU stay, day	0.4 ± 1.6	0.3 ± 0.8	1.4 ± 5.3	0.805	0.5 ± 2.5	0.2 ± 0.6	1.4 ± 5.3	0.028
Post-op hospital stay, day	5.8 ± 4.3	5.7 ± 3.6	6.9 ± 9.0	0.403	5.7 ± 5.6	5.4 ± 4.2	6.9 ± 9.0	0.205
Chest tube								
Chest tube duration, day	3.1 ± 3.1	3.2 ± 3.2	2.6 ± 1.9	0.542	2.9 ± 3.4	3.0 ± 3.7	2.6 ± 1.9	0.556
Chest tube ≥3 days	165 (43.1%)	152 (42.9%)	13 (44.8%)	0.843	51 (38.1%)	38 (36.2%)	13 (44.8%)	0.396
Chest tube >5 days	57 (14.9%)	54 (15.3%)	3 (10.3%)	0.596	16 (11.9%)	13 (12.4%)	3 (10.3%)	>0.999
Postoperative complications								
All complications	183 (47.8%)	163 (46.0%)	20 (69.0%)	0.018	66 (49.3%)	46 (43.8%)	20 (69.0%)	0.016
Grade 3a or greater	31 (8.1%)	29 (8.2%)	2 (6.9%)	>0.999	9 (6.7%)	7 (6.7%)	2 (6.9%)	>0.999
Grade 3b or greater	4 (1.0%)	2 (0.6%)	2 (6.9%)	0.030	2 (1.5%)	0 (0.0%)	2 (6.9%)	0.046
30-day mortality	0 (0.0%)	0 (0.0%)	0 (0.0%)	>0.999	0 (0.0%)	0 (0.0%)	0 (0.0%)	>0.999

Data are presented as mean ± standard deviation or number (%).

3DPA/Ao, Pulmonary artery/aorta diameter ratio measured on 3D images; ICU, intensive care unit; LN, lymph node; No, number; PA/Ao, ratio of the diameters of the pulmonary artery and aorta; Post-op, postoperative; VATS, video-assisted thoracoscopic surgery.

Clavien–Dindo classification showed significantly more postoperative complications in patients with 3D-measured PA/Ao ≥1 (69.0% *vs*. 46.0%; p = 0.018). Severe postoperative complications with grades greater than 3b were also more common in the 3D-measured PA/Ao ≥ 1 group. Four patients presented with postoperative complications above grade 3b: one patient developed empyema and required surgical decortication; one patient progressed to respiratory failure and underwent intubation and further tracheostomy; one patient developed pulmonary embolism complicated with pneumothorax and pneumomediastinum; and one patient had hemothorax and underwent reopening for stump ligation. Patients with complications of other grades are listed in **Supplementary Table 1**.

### Correlation between 3D and 2D measurements

The 3D-automated and 2D-manual arterial diameter measurements were closely related, with a significant correlation coefficient of 0.720 for the mean PA diameter and 0.780 for the mean Ao diameter (**Supplementary Figures 1, 2**). The classification by 2D measurement showed fewer patients in the PA/Ao ≥ 1 group than that by 3D measurement (13 and 29 patients). Other perioperative outcomes in the PA/Ao ≥ 1 and <1 groups categorized by 2D measurement are stated in **Supplementary Table 2**, none of which showed significant differences.

3D measurements of PA diameter and PA/Ao showed no significant dissimilarity in the patients with or without complications and patients with complications lower or greater than grade 3a. However, the 3D-measured PA/Ao was significantly higher in patients with postoperative complications greater than grade 3b (0.9 ± 0.1 *vs*. 0.8 ± 0.1; p = 0.8 ± 0.1). Specific diameters and ratios of PA and Ao are described in **Table 4**.

**Table 4:** PA measurements by automated 3D segmentation method relative to postoperative complications.

	All	Postoperative complications		p value
		None	All complication	
Main PA size, mm	27.8 ± 3.3	27.7 ± 3.2	28.0 ± 3.5	0.407
PA to AO ratio	0.8 ± 0.1	0.8 ± 0.1	0.8 ± 0.1	0.997
		Lower than grade 3a	Greater than grade 3a	
Main PA size, mm	27.8 ± 3.3	27.8 ± 3.4	27.6 ± 3.2	0.772
PA to AO ratio	0.8 ± 0.1	0.8 ± 0.1	0.8 ± 0.1	0.989
		Lower than grade 3b	Greater than grade 3b	
Main PA size, mm	27.8 ± 3.3	27.8 ± 3.3	30.1 ± 3.0	0.144
PA to AO ratio	0.8 ± 0.1	0.8 ± 0.1	0.9 ± 0.1	0.039

Data are presented as mean ± standard deviation or number (%).

AO, aorta; PA, pulmonary artery.

### Propensity-matched analysis

Before PSM, patients in the 3D-measured PA/Ao ≥ 1 group were younger (p = 0.001), had a lower Charlson comorbidity index (p = 0.029) and a higher possibility of receiving uniportal VATS (p = 0.055) than those in the 3D-measured PA/Ao < 1 group. Using a 1:4 matching ratio, PSM identified 29 to 105 well-balanced patients in PA/Ao ≥ 1 and <1 groups for comparison of postoperative complications. After PSM, no significant differences were observed in the patient demographics and tumor clinicopathological features. After matching, the group with PA/Ao ≥ 1 still showed more postoperative complications of all grades (p = 0.016) and grade 3b or greater (p = 0.046). Moreover, higher proportions of patients with cough or dyspnea, regarded as grade 2 complications, remained after matching (p = 0.005). Other detailed numbers before and after matching are described in **Tables 1**-**3**. The distribution of the propensity scores and standard mean difference for both groups before and after PSM is shown in **Supplementary Figure 3**.

### Correlations between clinical features and postoperative complications

In the multivariate analysis, age, sex, smoking status, Eastern Cooperative Oncology Group performance status, pulmonary function, Charlson comorbidity index, carcinoembryonic antigen value, histological type of lung cancer, differentiation of tumor, visceral pleural invasion, lymphovascular invasion, resection margin, and lung cancer staging did not affect the incidence of postoperative complications. Furthermore, only tumor size above 3 cm and 3D-measured PA/Ao ≥ 1 were associated with an increased risk of postoperative complications (odds ratios, 3.173 [p = 0.002] and 3.084 [p = 0.013], respectively) **Table 5**.

**Table 5:** Multivariate analysis between clinicopathologic features and postoperative complications (all grade) in this cohort.

Variable	Multivariate logistic regression		
	OR	95% CI	p value
Age			
<80 years	1.000		
≥80 years	1.597	0.123-20.683	0.720
Sex			
Male	1.000		
Female	0.898	0.510-1.579	0.708
Smoking status			
Never	1.000		
Current or former	0.994	0.495-1.995	0.987
ECOG performance status			
<1	1.000		
≥1	0.927	0.534-1.608	0.787
PFT^a^			
FEV_1_ < 80%	1.987	0.670-5.895	0.216
FEV_1_ ≥ 80%	1.000		
Comorbidity index (CCI)			
<5	1.000		
≥5	2.024	0.831-4.926	0.120
CEA^a^			
Normal	1.000		
Abnormal	1.582	0.771-3.244	0.211
Histologic type			
Non-adenocarcinoma	1.000		
Adenocarcinoma	0.334	0.063-1.774	0.198
Differentiation^a^			
Poor	1.000		
Well and moderate	0.766	0.363-1.616	0.483
VPI			
Negative	1.000		
Positive	1.907	0.773-4.703	0.161
LVI			
Negative	1.000		
Positive	0.547	0.267-1.125	0.101
Resection margin			
Negative	1.000		
Positive	2.523	0.844-7.543	0.098
Tumor size			
≤3 cm	1.000		
>3 cm	3.173	1.544-6.523	0.002
Stage			
1	1.000		
2-3	0.641	0.271-1.514	0.310
3D_PA to Ao ratio			
<1	1.000		
≥ 1	3.084	1.262-7.537	0.013

AO, aorta; CCI, Charlson comorbidity index; CEA, carcinoembryonic antigen; CI, confidence interval; ECOG, Eastern Cooperative Oncology Group; FEV1, forced expiratory volume in 1 second; FVC, forced vital capacity; LVI, lymphovascular invasion; OR, odds ratio; PA, pulmonary artery; PFT, pulmonary function test; VPI, visceral pleural invasion.

a Missing value for FVC/FEV1 (n = 10), CEA level (n = 28), and differentiation (n = 7).

## Discussion

Although lobectomy is considered the standard treatment for lung cancer, pulmonary hypertension (PH) remains a great challenge in preoperative risk assessments. We attempted to use the pulmonary artery/aorta (PA/Ao) diameter ratio with an automated 3D segmentation method on non-contrast CT scans to estimate PH (15). By matching patients with diameter ratios of ≥1 and <1, in terms of age, Charlson comorbidity score, and lobectomy performed by uniportal VATS we demonstrated that a larger PA diameter correlates with higher rates of overall postoperative complications and grade 3b or above severe complications. Multivariate analysis also revealed significant odds ratios for 3D-measured diameter ratio ≥ 1 and tumor size > 3 cm in predicting postoperative complications. Our 3D automated measurements showed a high correlation to manual annotation and demonstrated its usefulness in accurately predicting postoperative complications.

Although PH has been recognized as a comorbidity for patients undergoing anatomical lung resection, preoperative PH identification requires complicated diagnostic workups. Nevertheless, Ng et al. revealed that a PA/Ao diameter ratio > 1 indicated a high probability of PH (20). Multiple subsequent studies also identified the role of PA size in evaluating the prognosis of patients with chronic obstructive pulmonary disease and idiopathic pulmonary fibrosis (21, 22). In fact, a meta-analysis including 2134 subjects showed that the PA/Ao diameter ratio showed an area under the receiver operating characteristic value of 0.84 with median sensitivity and specificity of 74% and 81%, respectively, for predicting PH (23). Recent studies elaborated further on this topic by relating PA diameter to surgical complications by determining the PH through CT scans. Asakura et al. demonstrated an odds ratio of 2.3 for a 0.1-point increase in the PA/Ao ratio in predicting postoperative cardiopulmonary complications for patients undergoing pulmonary resection (10). Kneuertz simultaneously illustrated that an increase of 1 mm of surgical side PA yielded an odds ratio of 1.12 in predicting major complications for lobectomy patients (11). These findings correlated with our results, indicating that CT-based PA size evaluations could predict postoperative complications.

Although PH diagnosis based on CT scans is convenient, it still shows the inherent limitations associated with the use of 2D CT images to represent 3D geometry. Accordingly, a 3D segmentation method, which was previously applied for Ao, was developed to obtain accurate measurements of PA. Xie et al. first used 359 low-dose CT scans to develop an automated Ao segmentation model that showed a mean Dice similarity coefficient of 0.993 compared with the ground truth marking and an average boundary distance of 1.39 mm between manual and automated segmentation (24). Gamechi et al. further built an automated Ao segmentation model based on 100 CT scans and showed agreement with manual annotation for the Dice similarity coefficient, mean surface distance, and intra-class correlation (13). In both qualitative and quantitative evaluations, these two studies showed the plausibility of reconstructing 3D arterial morphology using flat CT images. Subsequently, this 3D segmentation technique was employed for PA measurements. Froelich et al. used a threshold-seeded vascular-tracing algorithm to reveal a high correlation between vascular volumes on CT and PA pressure, measured by right heart catheterization (with PH: Pearson correlation coefficient r = 0.90) (25). Additionally, Rengier et al. claimed an area under the receiver operating characteristic of 0.998 for predicting PH using PA volume on magnetic resonance angiography calculated by a 3D image analysis software (26). Nevertheless, unlike our study, which used PA diameter to determine PH, these studies utilized PA volume as an indicator, potentially hampering its practicality due to the non-intuitive technique nature.

Our study had several limitations. First, the inherent selection bias attributable to the retrospective nature could not be avoided. Although we tried to achieve a fairly random status through PSM, several noticeable differences still existed between patients with 3D-measured PA/Ao ≥ 1 and <1. Second, since this cohort only contained patients from a single center and a single surgical team, our result may not be generalizable to the entire population. Additional studies should be conducted on patients with different ethnicities or nations with distinguishable surgical considerations. Third, our 3D automated measurement method for PA and Ao was only validated in a relatively small population and was restricted to lobectomy patients. Additional investigations would be required to evaluate the performance on abnormal or tortuous artery shapes. However, since our method was an innovation based on previous literature and demonstrated outstanding prediction of postoperative complications, this technique could represent a cutting-edge change in the process of surgical planning.

In conclusion, higher overall and severe postoperative complications were observed in lobectomy patients with pulmonary artery/aorta diameter ratio ≥ 1 measured by our proposed 3D automated segmentation method. Moreover, the 3D-estimated diameter ratio and tumor size were significant predictors for postoperative complications. These findings may facilitate surgical planning and raise awareness for preoperative pulmonary hypertension assessment in patients with pulmonary artery enlargement.

## Data availability statement

The raw data supporting the conclusions of this article will be made available by the authors, without undue reservation.

## Ethics statement

The Research Ethics Committee of the National Taiwan University Hospital approved this study (project approval number 201712087RIND) and waived the need for obtaining informed consent because of the retrospective study design.

## Author contributions

Conception and design: H-JW, L-WC, Y-TL, H-HH, M-WL, J-SC; administrative support: H-HH, M-WL, J-SC; provision of study materials or patients: H-HH, Y-CC, C-MC, M-WL, J-SC; collection and assembly of data: H-YL, Y-JC, X-HC, and Y-CL; data analysis and interpretation: H-YL, Y-JC, X-HC, Y-CL, and M-WL; manuscript writing: all authors. All authors contributed to the article and approved the submitted version.

## Funding

This research was funded by the Ministry of Science and Technology, Taiwan (MOST 107-2221-E-002-074-MY3, 107-2221-E-002-080-MY3, 110-2314-B-002-271) and National Taiwan University Hospital, Taipei, Taiwan (NTUH 111-S0199).

## Conflict of interest

The authors declare that the research was conducted in the absence of any commercial or financial relationships that could be construed as a potential conflict of interest.

## Publisher’s note

All claims expressed in this article are solely those of the authors and do not necessarily represent those of their affiliated organizations, or those of the publisher, the editors and the reviewers. Any product that may be evaluated in this article, or claim that may be made by its manufacturer, is not guaranteed or endorsed by the publisher.

## References

[1] Port JL Mirza FM Lee PC Paul S Stiles BM Altorki NK . Lobectomy in octogenarians with non-small cell lung cancer: Ramifications of increasing life expectancy and the benefits of minimally invasive surgery. Ann Thorac Surg (2011) 92(6):1951–7. doi: 10.1016/j.athoracsur.2011.06.082 21982148

[2] Paul S Altorki NK Sheng S Lee PC Harpole DH Onaitis MW . Thoracoscopic lobectomy is associated with lower morbidity than open lobectomy: a propensity-matched analysis from the STS database. J Thorac Cardiovasc Surg (2010) 139(2):366–78. doi: 10.1016/j.jtcvs.2009.08.026 20106398

[3] Chiang XH Hsu HH Hsieh MS Chang CH Tsai TM Liao HC . Propensity-matched analysis comparing survival after sublobar resection and lobectomy for cT1N0 lung adenocarcinoma. Ann Surg Oncol (2019) 27:703–15. doi: 10.1245/s10434-019-07974-9 31646453

[4] Li C Kuo SW Hsu HH Lin MW Chen JS . Lung adenocarcinoma with intraoperatively diagnosed pleural seeding: Is main tumor resection beneficial for prognosis? J Thorac Cardiovasc Surg (2018) 155(3):1238–1249.e1. doi: 10.1016/j.jtcvs.2017.09.162 29254636

[5] Chen PH Hsu HH Yang SM Tsai TM Tsou KC Liao HC . Preoperative dye localization for thoracoscopic lung surgery: Hybrid versus computed tomography room. Ann Thorac Surg (2018) 106(6):1661–7. doi: 10.1016/j.athoracsur.2018.07.030 30218664

[6] Wei B D'Amico T Samad Z Hasan R Berry MF . The impact of pulmonary hypertension on morbidity and mortality following major lung resection. Eur J Cardiothorac Surg (2014) 45(6):1028–33. doi: 10.1093/ejcts/ezt495 PMC4020383 24132298

[7] Pierce RJ Sharpe K Johns J Thompson B . Pulmonary artery pressure and blood flow as predictors of outcome from lung cancer resection. Respirology (2005) 10(5):620–8. doi: 10.1111/j.1440-1843.2005.00759.x 16268916

[8] Ramakrishna G Sprung J Ravi BS Chandrasekaran K McGoon MD . Impact of pulmonary hypertension on the outcomes of noncardiac surgery: Predictors of perioperative morbidity and mortality. J Am Coll Cardiol (2005) 45(10):1691–9. doi: 10.1016/j.jacc.2005.02.055 15893189

[9] Hagiwara M Shimada Y Kato Y Nawa K Makino Y Furumoto H . High-quality 3-dimensional image simulation for pulmonary lobectomy and segmentectomy: Results of preoperative assessment of pulmonary vessels and short-term surgical outcomes in consecutive patients undergoing video-assisted thoracic surgery†. Eur J Cardiothorac Surg (2014) 46(6):e120–6. doi: 10.1093/ejcts/ezu375 25342848

[10] Asakura K Mitsuboshi S Tsuji M Sakamaki H Otake S Matsuda S . Pulmonary arterial enlargement predicts cardiopulmonary complications after pulmonary resection for lung cancer: a retrospective cohort study. J Cardiothorac Surg (2015) 9:10:113. doi: 10.1186/s13019-015-0315-9 PMC4564964 26353804

[11] Kneuertz PJ Yudovich MS Amadi CC Bashian E D'Souza DM Abdel-Rasoul M . Pulmonary artery size on computed tomography is associated with major morbidity after pulmonary lobectomy. J Thorac Cardiovasc Surg (2022) 163(4):1521–1529.e2. doi: 10.1016/j.jtcvs.2021.01.124 33685731

[12] Moyer VA Moyer VA LeFevre ML Siu AL Peters JJ Baumann LC . Screening for lung cancer: U.S. preventive services task force recommendation statement. Ann Intern Med (2014) 160(5):330–8. doi: 10.7326/M13-2771 24378917

[13] Sedghi Gamechi Z Bons LR Giordano M Bos D Budde RPJ Kofoed KF . Automated 3D segmentation and diameter measurement of the thoracic aorta on non-contrast enhanced CT. Eur Radiol (2019) 29(9):4613–23. doi: 10.1007/s00330-018-5931-z PMC6682850 30673817

[14] Sedghi Gamechi Z de Bruijne M Arias-Lorza AM Pedersen JH . Aorta and pulmonary artery segmentation using optimal surface graph cuts in non-contrast CT. In: ED A BA L , editors. Medical imaging 2018: Image processing, vol. p84. Houston, United States: SPIE (2018).

[15] Wang H-J Chen L-W Lee H-Y Chung YJ Lin YT Lee YC . Automated 3D segmentation of the aorta and pulmonary artery on non-contrast-enhanced chest computed tomography images in lung cancer patients. Diagnostics (2022) 12(4):967. doi: 10.3390/diagnostics12040967 35454015 PMC9032785

[16] Charlson ME Pompei P Ales KL MacKenzie CR . A new method of classifying prognostic comorbidity in longitudinal studies: Development and validation. J Chronic Dis (1987) 40(5):373–83. doi: 10.1016/0021-9681(87)90171-8 3558716

[17] Clavien PA Barkun J de Oliveira ML Vauthey JN Dindo D Schulick RD . The clavien-dindo classification of surgical complications: Five-year experience. Ann Surg (2009) 250(2):187–96. doi: 10.1097/SLA.0b013e3181b13ca2 19638912

[18] Travis WD Brambilla E Nicholson AG Yatabe Y Austin JHM Beasley MB . The 2015 world health organization classification of lung tumors: Impact of genetic, clinical and radiologic advances since the 2004 classification. J Thorac Oncol (2015) 10(9):1243–60. doi: 10.1097/JTO.0000000000000630 26291008

[19] Rami-Porta R Asamura H Travis WD Rusch VW . Lung cancer - major changes in the American joint committee on cancer eighth edition cancer staging manual. CA Cancer J Clin (2017) 67(2):138–55. doi: 10.3322/caac.21390 28140453

[20] Ng CS Wells AU Padley SP . A CT sign of chronic pulmonary arterial hypertension: the ratio of main pulmonary artery to aortic diameter. J Thorac Imaging (1999) 14(4):270–8. doi: 10.1097/00005382-199910000-00007 10524808

[21] Shin S King CS Puri N Shlobin OA Brown AW Ahmad S . Pulmonary artery size as a predictor of outcomes in idiopathic pulmonary fibrosis. Eur Respir J (2016) 47(5):1445–51. doi: 10.1183/13993003.01532-2015 26846836

[22] Iyer AS Wells JM Vishin S Bhatt SP Wille KM Dransfield MT . CT scan-measured pulmonary artery to aorta ratio and echocardiography for detecting pulmonary hypertension in severe COPD. Chest (2014) 145(4):824–32. doi: 10.1378/chest.13-1422 PMC3971971 24114440

[23] Shen Y Wan C Tian P Wu Y Li X Yang T . CT-base pulmonary artery measurement in the detection of pulmonary hypertension: a meta-analysis and systematic review. Med (Baltimore) (2014) 93(27):e256. doi: 10.1097/MD.0000000000000256 PMC4602811 25501096

[24] Xie Y Padgett J Biancardi AM Reeves AP . Automated aorta segmentation in low-dose chest CT images. Int J Comput Assist Radiol Surg (2014) 9(2):211–9. doi: 10.1007/s11548-013-0924-5 23877280

[25] Froelich JJ Koenig H Knaak L Krass S Klose KJ . Relationship between pulmonary artery volumes at computed tomography and pulmonary artery pressures in patients with- and without pulmonary hypertension. Eur J Radiol (2008) 67(3):466–71. doi: 10.1016/j.ejrad.2007.08.022 17913425

[26] Rengier F Wörz S Melzig C Ley S Fink C Benjamin N . Automated 3D volumetry of the pulmonary arteries based on magnetic resonance angiography has potential for predicting pulmonary hypertension. PloS One (2016) 11(9):e0162516. doi: 10.1371/journal.pone.0162516 27626802 PMC5023190

