# Supplementary material for: Automated 3D segmentation of the aorta and pulmonary artery for predicting outcomes after thoracoscopic lobectomy in lung cancer patients
Source: Front Oncol. 2022 Oct 28;12:1027036. doi: 10.3389/fonc.2022.1027036 (PMC9650405; doi:10.3389/fonc.2022.1027036)
Supplement: Supplementary file 1 [file Table_1.pdf]

Supplementary Table 1. Details of postoperative complications

	Before matching				After matching			
	All (n = 383)	3DPA/Ao<1 (n = 354)	3DPA/Ao≥1 (n = 29)	p value	All (n = 134)	PA/Ao<1 (n = 105)	PA/Ao≥1 (n = 29)	p value
**All complications**								
All complications	183 (47.8%)	163(46.0%)	20(69.0%)	0.018	66 (49.3%)	46 (43.8%)	20 (69.0%)	0.016
Grade 3a or greater	31 (8.1%)	29 (8.2%)	2 (6.9%)	>0.999	9 (6.7%)	7 (6.7%)	2 (6.9%)	>0.999
Grade 3b or greater	4 (1.0%)	2 (0.6%)	2 (6.9%)	0.030	2 (1.5%)	0 (0.0%)	2 (6.9%)	0.046
**Grade I**								
Subcutaneous emphysema	14 (3.7%)	11 (3.1%)	3 (10.3%)	0.081	8 (6.0%)	5 (4.8%)	3 (10.3%)	0.370
Pleural effusion	8 (2.1%)	6 (1.7%)	2 (6.9%)	0.117	5 (3.7%)	3 (2.9%)	2 (6.9%)	0.296
Others[a]	28 (7.3%)	26 (7.3%)	2 (6.9%)	>0.999	10 (7.5%)	8 (7.6%)	2 (6.9%)	>0.999
**Grade II**								
Urinary retention	5 (1.3%)	5 (1.4%)	0 (0.0%)	>0.999	0 (0.0%)	0 (0.0%)	0 (0.0%)	>0.999
Arrhythmia	9 (2.3%)	8 (2.3%)	1 (3.4%)	0.512	4 (3.0%)	3 (2.9%)	1 (3.4%)	>0.999
Pneumonia	6 (1.6%)	6 (1.7%)	0 (0.0%)	>0.999	1 (0.7%)	1 (1.0%)	0 (0.0%)	>0.999
Cough or dyspnea	15 (3.9%)	10 (2.8%)	5 (17.2%)	0.003	7 (5.2%)	2 (1.9%)	5 (17.2%)	0.005
GI discomfort	12 (3.1%)	11 (3.1%)	1 (3.4%)	>0.999	4 (3.0%)	3 (2.9%)	1 (3.4%)	>0.999
Prolong air leak	40 (10.4%)	37 (10.5%)	3 (10.3%)	>0.999	14 (10.4%)	11 (10.5%)	3 (10.3%)	>0.999
Pleural effusion	3 (0.8%)	3 (0.8%)	0 (0.0%)	>0.999	0 (0.0%)	0 (0.0%)	0 (0.0%)	>0.999
Chylothorax	4 (1.0%)	4 (1.1%)	0 (0.0%)	>0.999	1 (0.7%)	1 (1.0%)	0 (0.0%)	>0.999
Others[b]	8 (2.1%)	7 (2.0%)	1 (3.4%)	0.471	3 (2.2%)	2 (1.9%)	1 (3.4%)	0.522
**Grade IIIa**								
Prolong air leak	21 (5.5%)	21 (5.9%)	0 (0.0%)	0.389	5 (3.7%)	5 (4.8%)	0 (0.0%)	0.585
Pleural effusion	1 (0.3%)	1 (0.3%)	0 (0.0%)	>0.999	1 (0.7%)	1 (1.0%)	0 (0.0%)	>0.999
Chylothorax	3 (0.8%)	3 (0.8%)	0 (0.0%)	>0.999	1 (0.7%)	1 (1.0%)	0 (0.0%)	>0.999
Wound bleeding (suture)	2 (0.5%)	2 (0.6%)	0 (0.0%)	>0.999	0 (0.0%)	0 (0.0%)	0 (0.0%)	>0.999
**Grade IIIb**								
Decortication	1 (0.3%)	0 (0.0%)	1 (3.4%)	0.076	1 (0.7%)	0 (0.0%)	1 (3.4%)	0.216
Hemothorax (reoperation)	1 (0.3%)	1 (0.3%)	0 (0.0%)	>0.999	0 (0.0%)	0 (0.0%)	0 (0.0%)	>0.999
**Grade IVa**								
Pulmonary embolism	1 (0.3%)	1 (0.3%)	0 (0.0%)	>0.999	0 (0.0%)	0 (0.0%)	0 (0.0%)	>0.999
**Grade IVb**								
Respiratory failure	1 (0.3%)	0 (0.0%)	1 (3.4%)	0.076	1 (0.7%)	0 (0.0%)	1 (3.4%)	0.216
**Grade V**								
Death	0 (0.0%)	0 (0.0%)	0 (0.0%)	>0.999	0 (0.0%)	0 (0.0%)	0 (0.0%)	>0.999

Data are presented as mean ± standard deviation or number (percentage).

[a]Others include residual effusion after chest tube removal (n = 1), leg edema (n = 2), severe nausea and vomiting (n = 13), severe postoperative pain (n = 6), transient blood pressure change (n = 3), transient tachycardia (n = 1), transient skin rash (n = 1), transient chest tightness (n = 1)

[b]Others include pulmonary hypertension (n = 2), uncontrolled hypertension (n = 3), anorexia (n = 1), skin rash (n = 1), anemia (n = 1)

GI gastrointestinal

Supplementary Table 2. Perioperative outcomes of lung cancer patients undergoing lobectomy

	All (n = 383)	2DPA/Ao<1 (n = 370)	2DPA/Ao≥1 (n = 13)	p value
Comorbidity index (CCI)	2.3±1.7	2.3±1.7	2.2±1.9	0.636
Dissected LNs				
Total number	13.8±7.5	13.8±7.5	14.0±8.1	0.895
Total station	4.6±1.3	4.6±1.3	4.3±1.8	0.692
Operative bleeding, mL	41.9±103.8	42.8±105.3	15.3±37.5	0.310
Post-op ICU stay, day	0.4±1.6	0.4±0.9	2.3±7.7	0.826
Post-op hospital stay, day	5.8±4.3	5.7±3.6	8.0±13.5	0.247
Chest tube				
Chest tube duration, day	3.1±3.1	3.2±3.2	2.0±0.9	0.199
Chest tube ≥3 days	165 (43.1%)	160 (43.2%)	5 (38.5%)	0.732
Chest tube >5 days	57 (14.9%)	57 (15.4%)	0 (0.0%)	0.230
Postoperative complications				
All complications	183 (47.8%)	175 (47.3%)	8 (61.5%)	0.312
Grade 3a or greater	31 (8.1%)	30 (8.1%)	1 (7.7%)	>0.999
Grade 3b or greater	4 (1.0%)	3 (0.8%)	1 (7.7%)	0.129
30-day mortality	0 (0.0%)	0 (0.0%)	0 (0.0%)	>0.999

Data are presented as mean ± standard deviation or number (percentage).

CCI, Charlson comorbidity index; ICU, intensive care unit; LN, lymph node; No, number; Post-op, postoperative; VATS, video-assisted thoracoscopic surgery
