# Supplementary material for: Automated 3D segmentation of the aorta and pulmonary artery for predicting outcomes after thoracoscopic lobectomy in lung cancer patients
Source: Front Oncol. 2022 Oct 28;12:1027036. doi: 10.3389/fonc.2022.1027036 (PMC9650405; doi:10.3389/fonc.2022.1027036)

Supplementary figure 1 Correlation between 2D and 3D PA

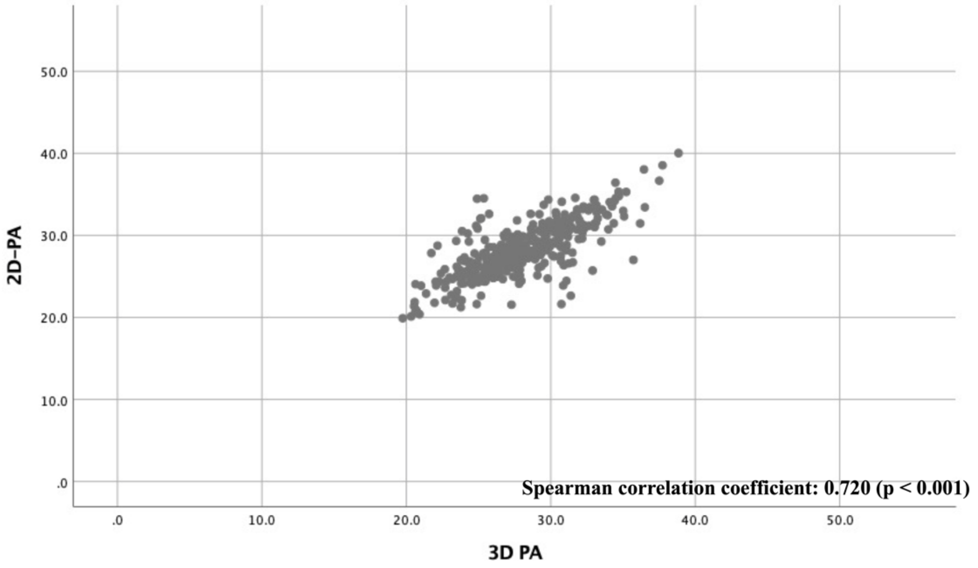

Supplementary figure 2 Correlation between 2D and 3D Ao

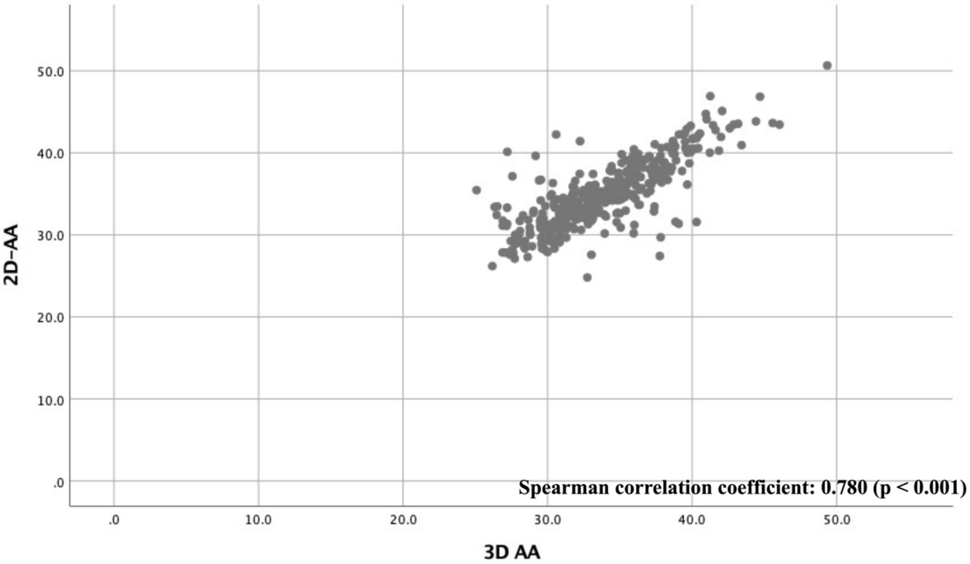

Supplementary Figure 3

Summary characteristics of the propensity score matching analysis.

(A) Distributions of propensity scores in both groups before and after matching.

(B) After matching, the two groups achieved minimal standardized mean differences in the matched variables.

(A)

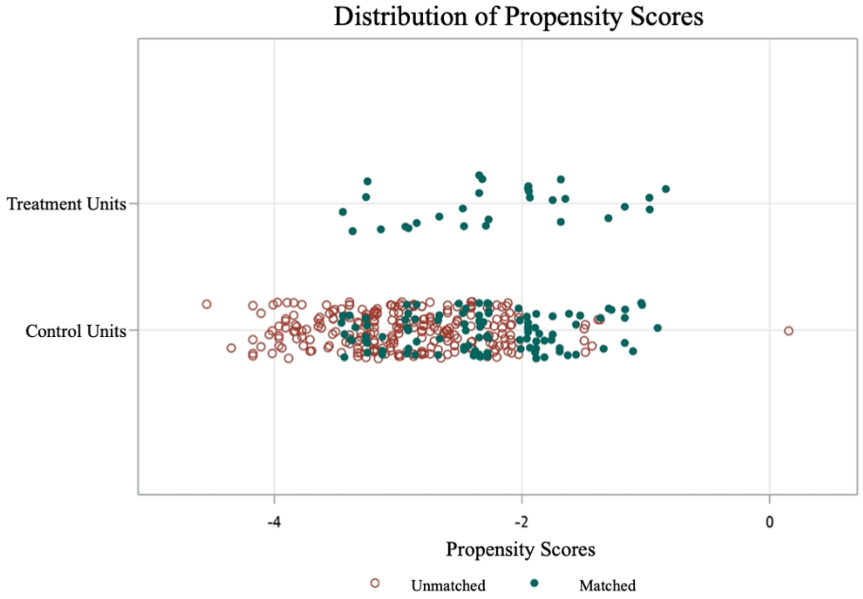

(B)

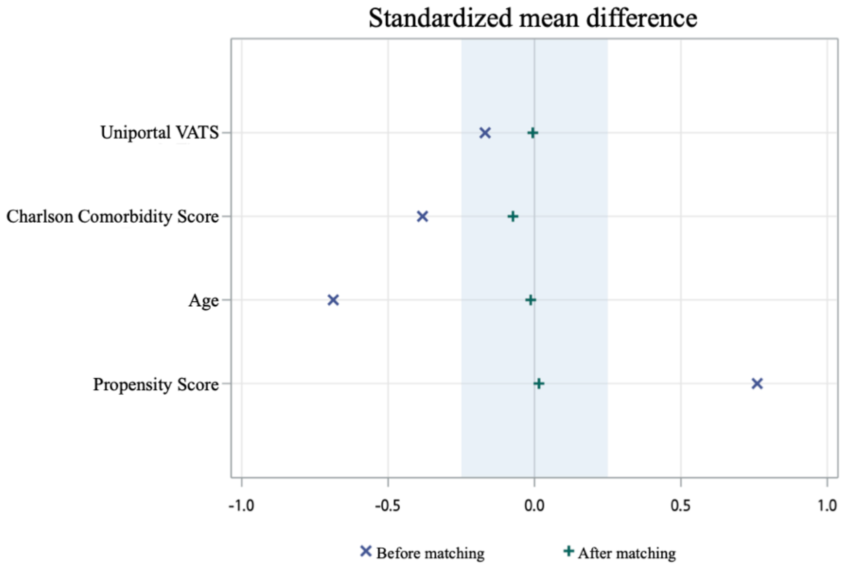

Supplement: Supplementary file 2 [file Image_1.pdf]
